# NMR evaluation of total statin content and HMG-CoA reductase inhibition in red yeast rice (*Monascus spp*.) food supplements

**DOI:** 10.1186/1749-8546-7-8

**Published:** 2012-03-22

**Authors:** Dirk W Lachenmeier, Yulia B Monakhova, Thomas Kuballa, Sigrid Löbell-Behrends, Sibylle Maixner, Matthias Kohl-Himmelseher, Asja Waldner, Christian Steffen

**Affiliations:** 1 Chemisches und Veterinäruntersuchungsamt (CVUA) Karlsruhe, Weissenburger Strasse 3, 76187 Karlsruhe, Germany; 2 Bruker Biospin GmbH, Silbersteifen, 76287 Rheinstetten, Germany; 3 Department of Chemistry, Saratov State University, Astrakhanskaya Street 83, 410012 Saratov, Russia; 4 Stabsstelle Ernährungssicherheit, Regierungspräsidium Tübingen, Konrad-Adenauer-Strasse 20, 72072 Tübingen, Germany; 5 Andreas-Schlüter-Strasse 19, 53639 Königswinter, Germany

## Abstract

**Background:**

Red yeast rice (*i.e*., rice fermented with *Monascus spp*.), as a food supplement, is claimed to be blood cholesterol-lowering. The red yeast rice constituent monacolin K, also known as lovastatin, is an inhibitor of the hydroxymethylglutaryl-CoA (HMG-CoA) reductase. This article aims to develop a sensitive nuclear magnetic resonance (NMR) method to determine the total statin content of red yeast rice products.

**Methods:**

The total statin content was determined by a 400 MHz ^1^H NMR spectroscopic method, based on the integration of the multiplet at δ 5.37-5.32 ppm of a hydrogen at the hexahydronaphthalene moiety in comparison to an external calibration with lovastatin. The activity of HMG-CoA reductase was measured by a commercial spectrophotometric assay kit.

**Results:**

The NMR detection limit for total statins was 6 mg/L (equivalent to 0.3 mg/capsule, if two capsules are dissolved in 50 mL ethanol). The relative standard deviations were consistently lower than 11%. The total statin concentrations of five red yeast rice supplements were between 1.5 and 25.2 mg per specified daily dose. A dose-dependent inhibition of the HMG-CoA reductase enzyme activity by the red yeast rice products was demonstrated.

**Conclusion:**

A simple and direct NMR assay was developed to determine the total statin content in red yeast rice. The assay can be applied for the determination of statin content for the regulatory control of red yeast rice products.

## Background

The fermentation products of *Monascus*, have been used as food and traditional Chinese medicine for over 1000 years [1]. The products are called "Hong Qu", "Hon-Chi", "Anka" or "Ang-kak" in China and Taiwan, "Beni Koji" or "red Koji" in Japan. In Europe, the products are called "red yeast rice", "red rice", "red mould rice" or "red Chinese rice". It should be noted that the designation "yeast" is taxonomically not correct [1]. Red yeast rice is used as an additive for the colouring, flavouring and preservation of foods, which may be permitted in some Asian countries but not in Europe. Currently red yeast rice products are predominantly marketed as food supplements, primarily sold through the Internet [2].

Monacolin compounds are formed by *Monascus *during the fermentation process. They cause a reversible competitive inhibition of the microsomal hydroxymethyl-glutaryl coenzyme A (HMG-CoA) reductase; thus, they prevent the reduction of HMG-CoA to mevalonic acid and the formation of cholesterol [2]. The major *Monascus *metabolite is monacolin K, which is structurally identical to lovastatin, the first statin drug introduced into the market [3,4]. The red yeast rice products that are currently marketed as food supplements differ from the traditional red yeast rice that is sold in Chinese groceries. The food supplements are manufactured using selected *Monascus *strains under carefully controlled and fully monitored conditions to increase the monacolin content [5].

The European Food Safety Authority (EFSA) has recently provided a scientific opinion on the health claims related to monacolin K from red yeast rice [6]. The opinion was based on two double-blind, placebo-controlled human intervention studies, which demonstrated a significant reduction in total cholesterol concentrations in the red yeast rice treatment groups compared to the placebo groups [7,8]. Besides these clinical trials, other studies also supported the efficacy of red yeast rice [9-11], as reviewed by Liu *et al. *[12]. A study regarding cholesterol synthesis in human hepatic cells *in vitro *showed that the inhibition of HMG-CoA reductase was facilitated by red yeast rice preparations, like lovastatin [13]. The monacolin K prepared from red yeast rice was effective for the maintenance of normal blood LDL-cholesterol levels [6]. As the EFSA [6] has not classified such products as food or medicine, the EFSA's opinion should not be interpreted as an approval of red yeast rice for food or medicinal use.

Red yeast rice is expected to have the adverse effects of its constituent lovastatin, including an increased risk of myopathy [14-18], acute rhabdomyolysis [19], symptomatic hepatitis [20] and anaphylactic reactions [21]. Drug interactions may influence liver enzyme expression leading to a lower clearance and elevated plasma concentration of lovastatin (*e.g*., by inhibition of cytochrome P450 enzyme CYP3A4) [19,22]. When lovastatin is administered with food, the intestinal absorption may be increased by about 50%, while pectin and oat bran may decrease its absorption [22-24]. The excessive use of grapefruit juice may inhibit CYP3A4 and lead to higher plasma concentrations of lovastatin [25,26].

Accurate determination of the statin content in red yeast rice products would be necessary for the regulatory control. Previous analytical approaches mainly used high-performance liquid chromatography (HPLC) with diode array or mass spectrometric detectors [27-33]. Song *et al. *[34] suggested a flow injection tandem mass spectrometry for screening analysis. Chromatographic and mass spectrometric methods have some problems in separating over 14 monacolins and avoiding co-eluting interferences that could cause overestimation of the quantities of the analytes [34]. Furthermore, an equilibrium exists between monacolin K and its lactone ring opened hydroxy acid form (ratio between 2:1 and 3:2), which can be chromatographically separated [32]. For this reason, the total monacolin content should be determined for the evaluation of such products [28,32,35]. The exclusive determination of the lactone form of monacolin K could underestimate the pharmacological activity. Accurate quantification for the hydroxy acid and other monacolin isomers was difficult because of the lack of commercial reference standards [34].

In this study, we overcome these problems by nuclear magnetic resonance (NMR) spectroscopy, which is advantageous for quantitative pharmaceutical analysis due to its high selectivity [36]. Dependent on the selected chemical shifts for quantification, such as protons at the hexahydronaphthalene moiety common to all first generation statins [37], the determination of total statins appears to be feasible with NMR without reference standards for each specific compound. A commercial HMG-CoA reductase assay kit was used to confirm the effects of the red yeast rice. The samples purchased over the Internet were evaluated by these methods.

## Materials and methods

### Samples and sample preparation

An internet search was conducted in November 2011 to identify the red yeast rice products that were offered at German speaking websites to German speaking consumers (for exact methodology, see Löbell-Behrends *et al. *[38,39]). None of the identified products was available in conventional retail stores. We purchased five different products, all were marketed to German consumers as food supplements in capsule form (*i.e*., the red yeast rice is provided in powder form inside a capsule).

For sample preparation, the content of two capsules (about 0.9 g) was dissolved in 50 mL of absolute ethanol (Merck, Darmstadt, Germany). An aliquot of 170 μL of this solution was mixed with 370 μL of distilled water and 60 μL of pH 7.4 NMR buffer (1.5 M KH_2_PO_4 _in D_2_O, 0.1% 3-(trimethylsilyl)-propionate acid-d_4 _(TSP), 3 mM NaN_3_). The mixture was poured into an NMR tube and directly measured. A lovastatin (Teva, Debrecen, Hungary) stock solution (500 mg/L) was prepared in absolute ethanol. For calibration, standards were prepared by diluting the lovastatin stock solution with ethanol (40% vol).

### NMR Method

All ^1^H NMR measurements were performed using a Bruker Avance 400 Ultrashield spectrometer (Bruker BioSpin, Rheinstetten, Germany) equipped with a 5-mm SEI probe with Z-gradient coils and an Automatic Sample Changer B-ACS 120 (Bruker BioSpin, Rheinstetten, Germany). All spectra were acquired at 300.0 K.

The NMR method was modified from our previous work for testing other products [40]. Two successive ^1^H NMR experiments were used for the measurement of each sample. First, the standard Bruker BioSpin water pre-saturation pulse program ZGPR was used to suppress only the signal of OH-protons. The relaxation delay (RD), and acquisition time (AQ) were set to 4 s and 3.99 s, respectively, resulting in a total recycle time of 7.99 s. After application of four dummy scans (DS), eight free induction decays (FIDs) (number of scans, NS = 8) were collected into a time domain (TD) of 65536 (65 k) complex data points using a 20.5187 ppm spectral width and a receiver gain of 1. The FIDs were multiplied with an exponential function corresponding to a line broadening of 1 Hz prior to Fourier transformation. Second, 8-fold suppression of water and ethanol was performed with the frequencies identified in the first experiment (Bruker sequence NOESYGPPS1D). The settings for the parameters RD, P(90°), AQ, and TD were kept similar to the ones from the first experiment, DS = 4 and NS = 32 were used and the mixing time (t_m_) was set to 10 ms.

The data were acquired automatically under the control of ICON-NMR (Bruker BioSpin, Rheinstetten, Germany), requiring about 12 min per sample. All NMR spectra were phased, baseline-corrected and integrated using Topspin 3.1 (Bruker BioSpin, Rheinstetten, Germany). For quantification, linear calibration curves were constructed from the lovastatin standards by integrating the multiplet at δ 5.37-5.32 ppm against TSP as an intensity reference. All measurements were done in five replicates including sample preparation. For ^1^H NMR spectral prediction, the software ChemBioDraw 12.0 (CambridgeSoft, Cambridge, UK) was used. The spectral prediction was performed according to Schaller *et al. *[41].

### Validation of NMR method

For method validation, standard solutions and authentic red yeast rice samples were analyzed several times on one day (intraday, n = 5) and over five consecutive days (interday, n = 10). The linearity of the calibration curves was evaluated in the range covering the concentrations found in the investigated products. The recovery rate was ascertained by adding lovastatin standard solution at two different concentrations to a real sample. The limits of detection (LOD) and quantification (LOQ) were calculated from the residual standard deviation of the regression line [42].

### HMG-CoA reductase assay

The HMG-CoA reductase (HMGR) assay kit #CS1090 from Sigma-Aldrich (Saint Louis, MO, USA) was used. The procedure was modified from Perchellet *et al. *[43], and according to the manufacturer's instructions, with the exception of an additional dilution 1:3 in assay buffer to facilitate the use of standard 1 cm cuvettes (*i.e*., 3 mL final volume instead of 1 mL volume). The inhibitor solution (pravastatin) provided with the kit was used as positive control.

The red yeast rice sample solutions prepared for NMR analysis were used for the assay in appropriate dilution. Spectrophotometric measurements were performed on a Perkin Elmer Lambda 20 dual beam spectrometer (Perkin Elmer, Rodgau, Germany) at 37°C. The spectrometer was operated with the Perkin Elmer UV WinLab software (version 2.85.04) in time drive mode. The absorbance at 340 nm was monitored at a time interval of 1.00 s for a total time of 15 min. The slit width was 1.00 nm and the response time 0.1 s.

### Statistical methods

All data were evaluated using Origin V.7.5 (OriginLab, Northampton, USA). Data are presented as means and standard deviations among replicates. Linear regression analysis was used to compare NMR responses and concentrations. *P *< 0.05 was considered statistically significant.

## Results and discussion

### NMR quantification results

**Figure 1:**
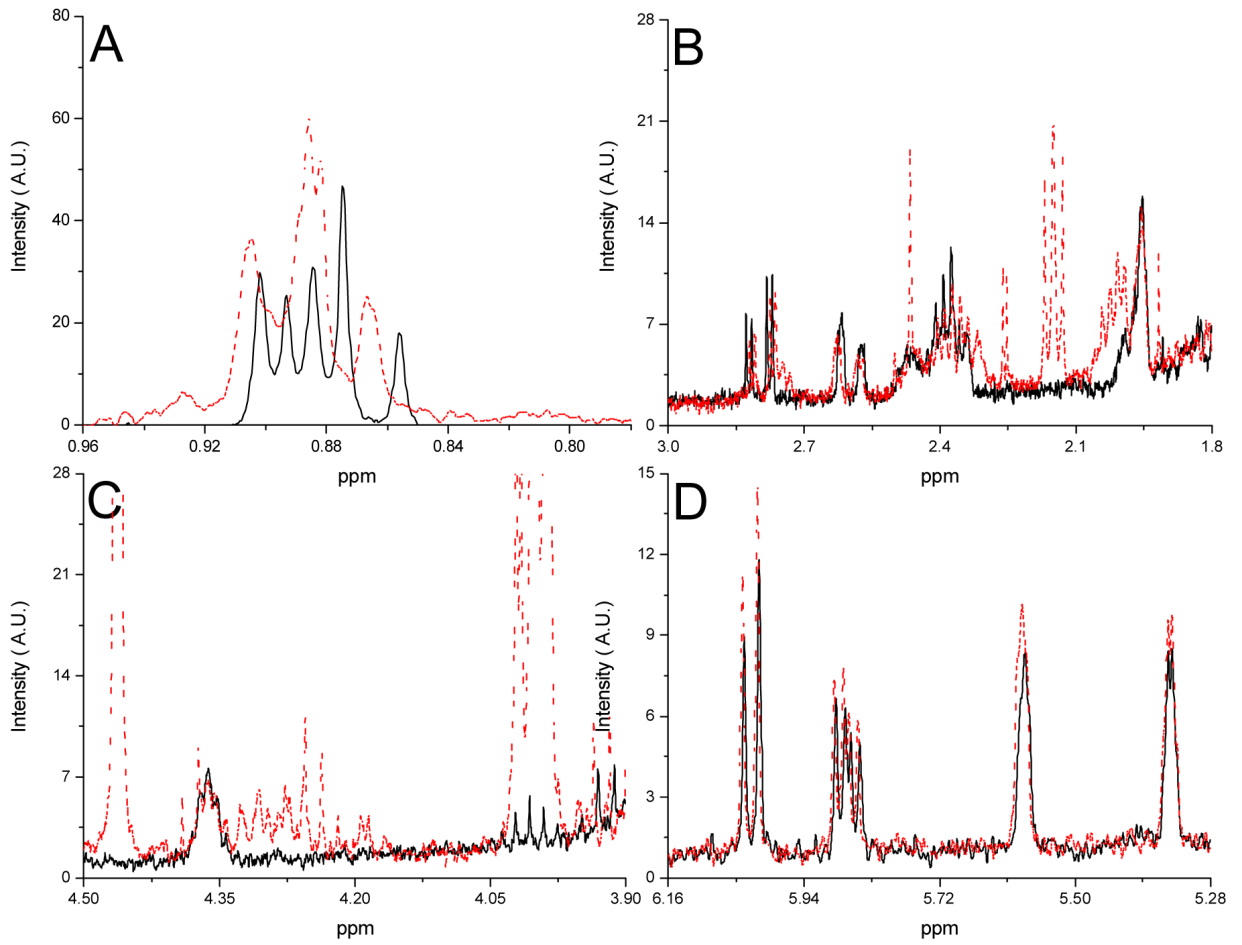
**400 MHz ^1^H NMR spectra of lovastatin standard (50 mg/L, solid line) compared with a red yeast rice product (sample S4, dashed line)**.

NMR determination of lovastatin showed resonances at δ 6.05-6.01 ppm, δ 5.90-5.84 ppm, δ 5.61-5.56 ppm, δ 5.37-5.32 ppm, δ 4.39-4.34 ppm (mid-field region) and δ 2.85-2.76 ppm, δ 2.63-2.56 ppm, δ 2.44-2.33 ppm, δ 1.98-1.93 ppm, δ 1.64-1.57 ppm, δ 0.91-0.85 ppm (aliphatic range) through comparing the spectra of lovastatin standard solutions with the spectra of authentic red yeast rice samples (Figure 1). However, the resonances in the aliphatic range were unsuitable for quantification because they showed strong overlap with matrix compounds (Figure 1A, B). Considering signals in the mid-field region (Figure 1C, D), we used the multiplet at δ 5.37-5.32 ppm for quantification because this led to the best sensitivity and this signal was not interfered in any case in our samples. Thus, more advanced techniques, such as multivariate regression or curve deconvolution, were not required.

**Figure 2:**
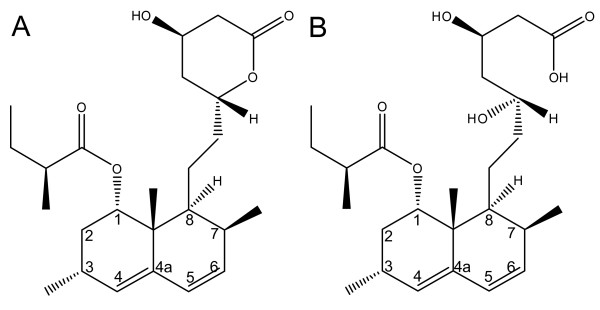
**Lovastatin (monacolin K) may occur in red yeast rice as lactone (A) or hydroxy acid (B)**.

A spectral assignment of lovastatin was provided by Chen *et al. *[44] at 600 MHz in D-methanol, by Holzgrabe *et al. *[45,46] at 300 MHz in CDCl_3_, by Ahmad *et al. *[47] at 300 MHz in DMSO-d_6_, and by Lankhorst *et al. *[48] at 600 MHz in CDCl_3_. While our conditions are not directly comparable to any of these studies, the literature data along with own spectral prediction show that the multiplet at δ 5.37-5.32 ppm clearly belongs to an H-atom at the hexahydronaphthalene moiety (Figure 2), most probably H6, H4 or an overlap of the signals of both atoms. A final spectral assignment would necessitate further 2D experiments outside the scope of this work. The use of a chemical shift belonging to the hexahydronaphthalene moiety has the advantage that not only the sum of the lactone and hydroxy acid forms of monacolin K (lovastatin) can be quantified, but a sum of all statins that contain the hexahydronaphthalene moiety, including monacolins J, L, M, or X in red yeast rice [1,29,49] and other first generation statins (*e.g*., used as additives to food supplements) but not second generation synthetic statins [37]. We therefore referred the results of our method to "total statins", not lovastatin or monacolin content.

Table 1 summarizes the NMR method validation results for lovastatin. The ^1^H NMR assay was linear in a working concentration range of 5-100 mg/L (R = 0.98, *P *< 0.0001). The LOD and LOQ were 6 and 13 mg/L, respectively, which were equal to 0.33 and 0.72 mg/capsule if two capsules were dissolved in 50 mL ethanol. These results were suitable for estimating the levels of lovastatin in red yeast rice products. The method was further validated by repeated sample preparations of standard samples and authentic red yeast rice samples. For the standard solutions, all relative standard deviations (RSD) were below 8% (intraday) and 9% (interday). For the authentic samples, the RSDs were slightly higher but still below 11%. The method validation result shows that the method is sufficiently precise and reproducible and can be used for analysis of authentic samples (Table 1).

**Table 1:** Results of method validation for monacolin K (lovastatin) using ^1^H NMR

Parameter	Result
Investigated linear working range	5 - 100 mg/L
Limit of detection^a^	6 mg/L (0.33 mg/capsule^c^)
Limit of quantitation^a^	13 mg/L (0.72 mg/capsule^c^)
Intraday precision^b^	
Authentic sample^d^	10%
Standard solution	7.3%
Interday precision^b^	
Authentic sample^d^	11%
Standard solution	8.2%
Recovery	104% (at 50 mg/L) 113% (at 20 mg/L)

^a ^Limit of detection (LOD) and quantitation were determined by establishing a separate calibration curve near LOD. The limits were calculated from the residual standard deviation of the regression line [42]

^b ^Precisions were expressed as relative standard deviation (%), intraday (n = 5), interday (n = 10)

^c ^Based on a sample weight of 0.9 g (2 capsules) in 50 mL ethanol

^d ^Total statins in authentic samples

**Figure 3:**
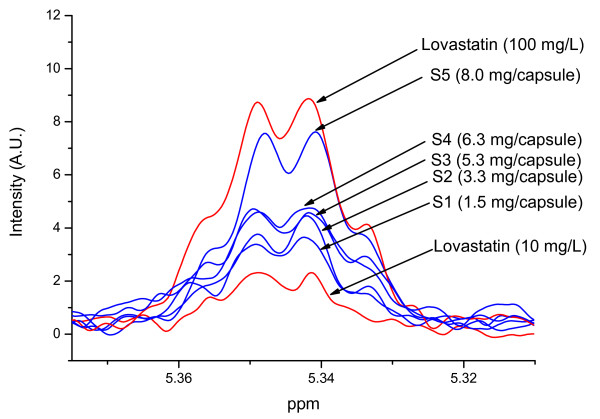
**400 MHz ^1^H NMR spectra of pure lovastatin and red yeast rice samples (S1-S5)**.

Five authentic red yeast rice products were analyzed by the validated method. Figure 3 shows the NMR peaks of integration from all analyzed products (S1-S5) in comparison with two reference spectra of lovastatin (10 and 100 mg/L). According to the ^1^H NMR data, all five analyzed samples contained statins ranging from 1.5 mg/capsule (S1) to 8.0 mg/capsule (S5) (Table 2). The results were in agreement with the manufacturers' labelling for two of the products (S1 and S5; for the other three products no labelling was provided). The determined concentrations correspond to a range between 1.5 and 25.2 mg per specified daily dose. Three of five investigated products (S3-5) had a dose higher than the starting dosage of prescription (10 mg per day) in humans [50-52]. Our results confirmed the previous studies that there is a wide variability in total statin content among different red yeast rice products [28,35] as well as a lack in product standardization [33]. Four out of the five red yeast rice products analyzed in this study failed to comply with the current European Union food labelling and food supplements regulations.

**Table 2:** Results of the quantitative ^1^H NMR determination of total statins in red yeast rice products

Sample	Sample Labelling	Product origin	Total statins (NMR)		Labelling of monacolin K (mg/capsule)
			(mg/capsule)^a^	(mg/daily dose)^b^	
S1	Red rice, food supplement, 330 mg capsules (in German)	Austria	1.5 ± 0.1	1.5 - 4.5	1.33
S2	Red yeast rice, food supplement, 600 mg capsules (in English)	USA with UK labelling	3.3 ± 0.4	6.6	no labelling
S3	Red yeast rice, herbal supplement, 330 mg capsules (in English)	UK	5.3 ± 0.6	10.6	no labelling
S4	Red yeast rice, dietary supplement, 600 mg capsules (in English)	USA	6.3 ± 0.2	12.6 - 25.2	no labelling
S5	Red yeast rice, food supplement, 600 mg capsules (in French)	France	8.0 ± 0.6	16.0 - 24.0	9.6

^a ^Means ± standard deviations (SD) based on 5 replicated measurements (including sample preparation)

^b ^The daily doses were calculated according to our analytical result and the intake recommendation of the products (a range is given when the manufacturer recommended a range for daily dose)

### HMG-CoA reductase inhibition

**Figure 4:**
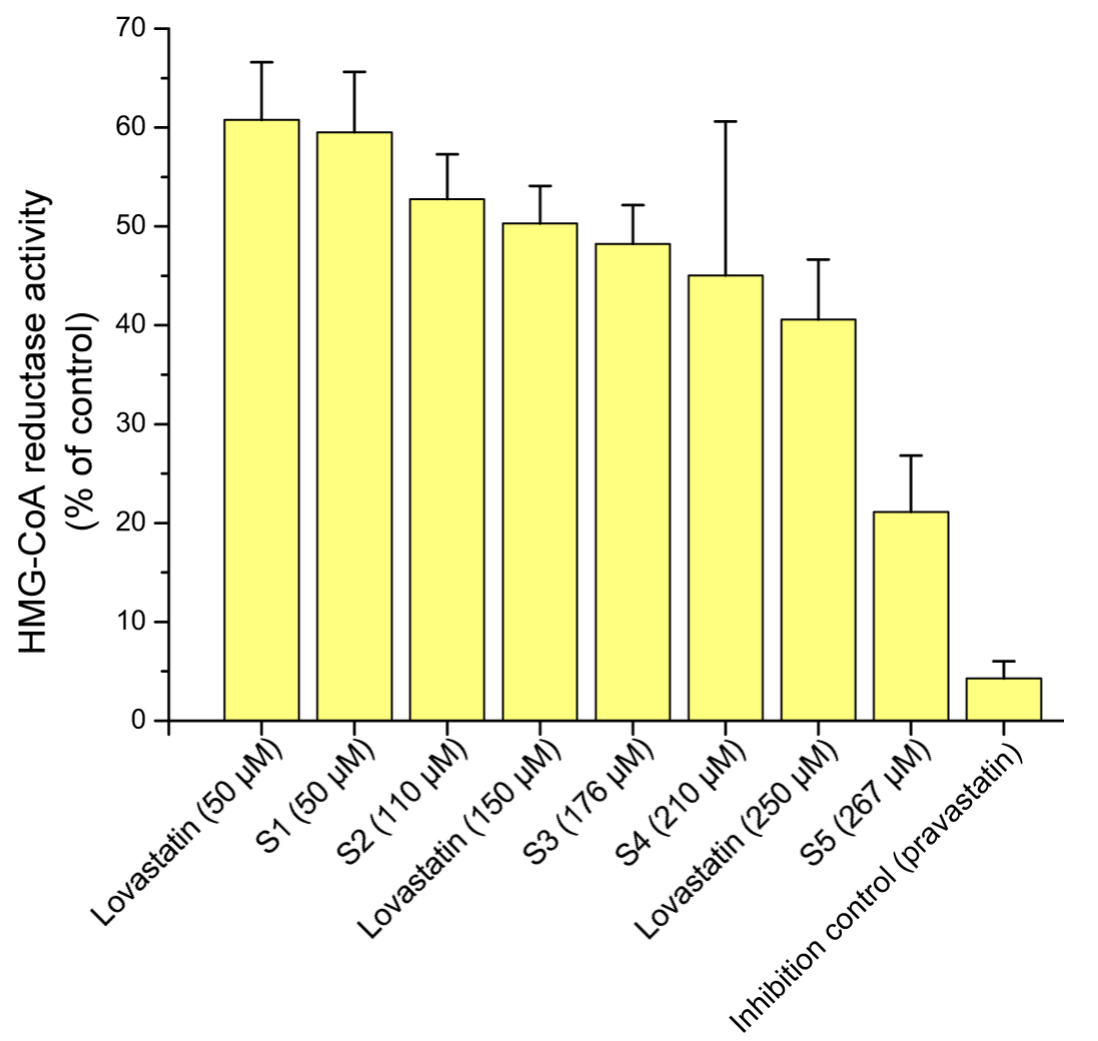
***In vitro *inhibition of human HMG-CoA reductase at increasing concentrations of monacolins in commercial red yeast rice product and pure lovastatin**. Results are expressed as % of the respective control specific activity of the enzyme in the absence of inhibitor (mean of two replicates; error bar: standard deviation).

Besides monacolin K, red yeast rice may contain several different monacolins with statin-like activity. Figure 4 shows that the activities were almost congruent (*e.g.*, compare lovastatin at 50 μM and S1 at 50 μM) when the total statin content in a red yeast rice sample was adjusted to the same molarity as a lovastatin standard. The inhibitory effect on HMG-CoA reductase was visually clear in Figure 4 to be related to the doses. In other words, the higher was the concentration of total statins in the red yeast rice, the greater the inhibitory effect exhibited. These results suggest that NMR can be used to determine the content of statins that would have inhibitory effect on HMG-CoA reductase.

## Conclusion

A simple and direct NMR assay was developed to determine the total statin content in red yeast rice. The assay can be applied for the determination of statin content for the regulatory control of red yeast rice products.

## Competing interests

The authors declare that they have no competing interests.

## Authors' contributions

DWL conceived of the study, coordinated the work, and wrote the manuscript. YBM carried out the NMR experiments including data acquisition and analysis, prepared the tables and figures, and wrote the materials and methods section of the manuscript. TK supervised the NMR work and revised the manuscript. SLB conducted the Internet search, purchased the products and revised the manuscript. SM conducted the checking of the food labelling of the products. SM, MKH and AW contributed knowledge about the regulatory control of the products and revised the manuscript. CS conceived of the study on the red yeast rice products sold on the Internet and the use of the HMG-CoA reductase assay, contributed to writing the introduction and discussion sections, and revised the manuscript. All authors read and approved the final manuscript.
